# An Unexpected Diagnosis Presenting as Hip Pain After a Fall

**DOI:** 10.5811/cpcem.2017.7.35306

**Published:** 2017-10-03

**Authors:** Jeremy Sauer, Eric Morgan

**Affiliations:** Virginia Commonwealth University, Department of Emergency Medicine, Richmond, Virginia

## CASE PRESENTATION

**Image 1:**
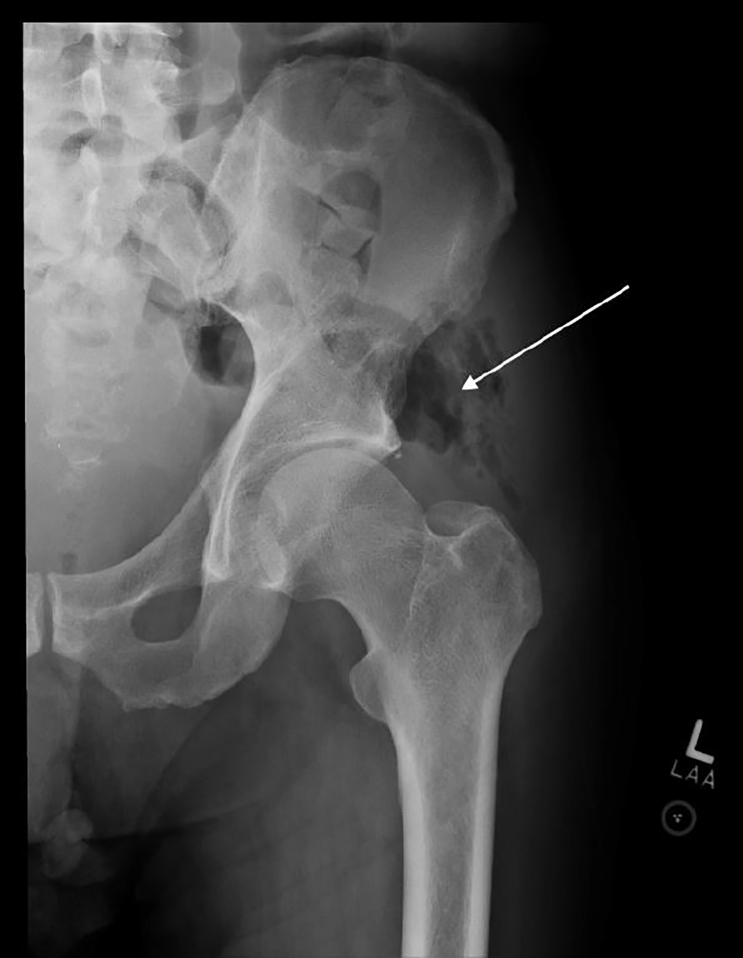
Anterior-posterior left hip radiograph demonstrating free air (arrow) overlying the left acetabulum.

**Image 2:**
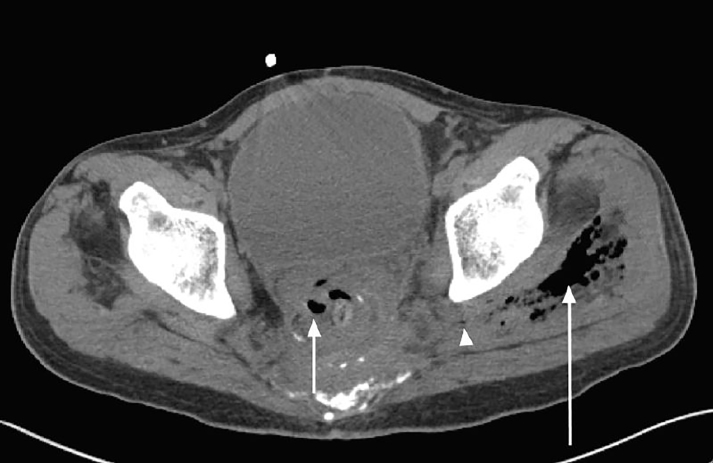
Axial computed tomography of the abdomen and pelvis demonstrating air and fluid in the pelvis (short arrow), exiting the sciatic notch (arrowhead) into the left buttock, with a large air and fluid collection centered in the left middle gluteal muscle (long arrow).

A 62 year old male presented to the emergency department with a complaint of two weeks of isolated left hip pain after slipping down two stairs three weeks prior to presentation. Initially well, the patient began experiencing progressive pain with ambulation. The patient’s history was significant for recurrence of rectal adenocarcinoma treated by surgical resection 10 years prior. On arrival to the emergency department, the patient ambulated with an antalgic gait. He was tachycardic (100–110 beats per minute), but vital signs were otherwise normal. Physical exam revealed bilateral lower extremity edema (left greater than right), left lateral hip tenderness, and painful range of motion of the left hip. The clinician’s primary concern was for left hip fracture which prompted a work up including left hip radiographs. The left hip radiographs revealed air within the soft tissues overlying the left acetabulum, best seen on the anterior-posterior view (Image 1). This prompted a non-contrast CT (Computed Tomography) scan (due to iodine allergy) that demonstrated free air and fluid coming from a perforated rectum, just superior to the rectal anastomosis, exiting through the sciatic notch, and forming an abscess in the left middle gluteal muscle (Image 2). Given these CT findings, intravenous antibiotics were started, and the patient was admitted for a laparoscopic descending end colostomy with two pelvic gluteal drains placed. During surgery, adenocarcinoma was confirmed as the culprit for his perforated rectum. The patient was discharged 23 days later after an uncomplicated hospitalization.

## DISCUSSION

With air seen on plain radiographs of the hip, additional differential diagnoses including septic arthritis, soft tissue infections from gas-forming organisms, and bowel perforations should be considered. An undiagnosed rectal perforation/gluteal abscess may lead to complications including peritonitis, necrotizing fasciitis, or sepsis.^1^ ^2^ With an abnormal radiograph, additional CT imaging including the abdomen and pelvis may be warranted.

**CPC-EM Capsule**

What do we already know about this clinical entity?

Air found in or near a joint on a plain radiograph is an ominous finding. Differential diagnoses to consider generally include septic arthritis or soft tissue infection from gas-forming pathogens.

What is the major impact of the images?

We present an image that lead to an unexpected diagnosis of bowel perforation after air was discovered at the hip joint. This image was obtained while investigating what seemed to be an orthopedic injury.

How might this improve emergency medicine practice?

This case reminds us that free air on plain radiograph is nearly always an ominous finding that requires additional investigation which may include advanced imaging. It is important to keep an open mind, especially when we are surprised by findings that don’t meet our clinical expectations.
